# Density-functional study of pressure-induced phase transitions and electronic properties of Zn_2_V_2_O_7_

**DOI:** 10.1039/d1ra01413g

**Published:** 2021-03-10

**Authors:** Daniel Díaz-Anichtchenko, Lourdes Gracia, Daniel Errandonea

**Affiliations:** Departamento de Física Aplicada-ICMUV, Universidad de Valencia Dr. Moliner 50, Burjassot 46100 Valencia Spain daniel.errandonea@uv.es; Departamento de Química Física, Universidad de Valencia Dr. Moliner 50, Burjassot 46100 Valencia Spain

## Abstract

We report a study of the high-pressure behavior of the structural and electronic properties of Zn_2_V_2_O_7_ by means of first-principle calculations using the CRYSTAL code. Three different approaches have been used, finding that the Becke–Lee–Yang–Parr functional is the one that best describes Zn_2_V_2_O_7_. The reported calculations contribute to the understanding of previous published experiments. They support the existence of three phase transitions for pressures smaller than 6 GPa. The crystal structure of the different high-pressure phases is reported. We have also made a systematic study of the electronic band-structure, determining the band-gap and its pressure dependence for the different polymorphs. The reported results are compared to previous experimental studies. All the polymorphs of Zn_2_V_2_O_7_ have been found to have a wide band gap, with band-gap energies in the near-ultraviolet region of the electromagnetic spectrum.

## Abstract 2

Phase transitions induced by pressure in zinc pyrovanadate have been understood using density-functional theory calculations. Consequences of structural changes on electronic properties are discussed.

## Graphical abstract

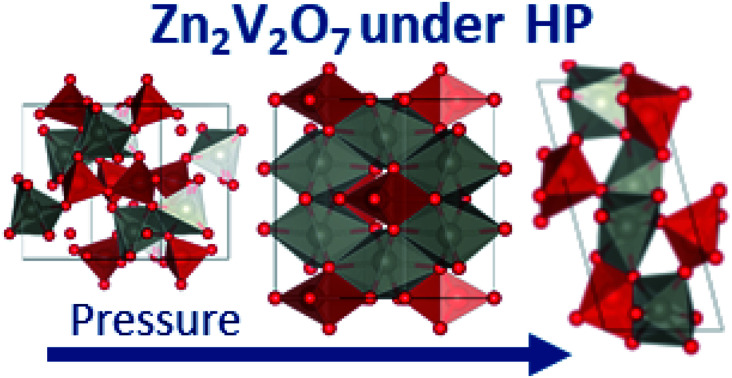

## 1. Introduction

Zinc pyrovanadate (Zn_2_V_2_O_7_) and related compounds are receiving a great deal of attention because of the multiple practical applications of these compounds. The applications include hydrogen storage,^1^ photocatalytic water splitting,^2^ batteries,^3^ and supercapacitors,^4^ among others. These vanadates not only have optimal optical and electronic properties for such uses, but also are chemically stable under mild alkaline conditions. As a consequence, their properties are being currently systematically studied.^1–8^ In addition to technological applications, Zn_2_V_2_O_7_ has recently raised attention because of its interesting behavior under high-pressure conditions.^8^ In particular, it has been reported that Zn_2_V_2_O_7_ (with a bulk modulus of 58 GPa (ref. 7)) is extremely more compressible that related vanadates like ZnV_2_O_6_ and Zn_3_V_2_O_8_ (with bulk moduli of 129 and 120 GPa, respectively).^7^ Not only that, differences between the compressibility of Zn_2_V_2_O_7_ and other vanadates also affect the structural stability under compression. According to X-ray diffraction experiments, Zn_2_V_2_O_7_ experiences three structural phase transitions under relative small compression (pressure < 11 GPa), while in the other compounds there are no phase transitions in the same pressure range.^7^ In particular, in Zn_2_V_2_O_7_ the transition from the ambient-pressure monoclinic polymorph (α-phase) to another monoclinic polymorph (β-phase) occurs at 1.1 GPa (ref. 7) and subsequent transitions take place at 3.8 GPa) (to a triclinic polymorphs named as γ-phase) and 10.8 GPa (post-γ-phase).^7^ For two of the high-pressure (HP) polymorphs (β and γ) a crystal structure has been proposed from previous X-ray diffraction studies; however, the crystal structures have not been properly solved.^7^ The proposed structures involve large changes in the unit-cell volume and a modification of the coordination polyhedra. Therefore, they are expected to affect other physical properties, in particular, the electronic band gap.^8^ However, the influence of high-pressure on the electronic properties of Zn_2_V_2_O_7_ has not been studied yet. Thus additional HP studies on Zn_2_V_2_O_7_ are relevant and timely.

Density-functional theory (DFT) calculations have proven to be a quite efficient tool to study the HP behavior of ternary oxides, and in particular vanadates.^9–12^ In this work, we will use this technique to examine the existence of pressure-driven phase transitions in Zn_2_V_2_O_7_. By considering the previously proposed HP crystal structures^7^ for this compound and other candidate structures, we have obtained a structural sequence, which will be compared to previous experiments.^7^ In addition, to crystallographic information on the different structures, we will also report their compressibilities and equations of state as well as their band structures and electronic densities of states. The band-structure calculations here reported support that Zn_2_V_2_O_7_ is a wide band-gap material, clarifying discrepancies found in the literature with values for band-gap energy (*E*_g_) going from 2.5 to 3.5 eV.^2,13–16^

## 2. Computational details

First-principles total-energy calculations have been carried out within the periodic DFT framework using the CRYSTAL14 program package.^17^ The applied density-functional approximations were the popular Becke–Lee–Yang–Parr (B3LYP)^18,19^ and Heyd–Scuseria–Ernzerhof (HSE06)^20^ hybrid functionals as well as the widely used Perdew–Burke–Ernzerhof (PBE) functional.^21^ For the calculations, Zn, V, and O atoms have been described by 86-4111d41G, 86-411d3G, and 6-31d1G all electron basis sets, respectively, which were taken from the Crystal website.^22^

The candidate structures are those proposed in the previous experimental study^7^ and potential structures selected according to crystal-chemistry arguments:^23^ α-Zn_2_V_2_O_7_ (space group (S.G.) *C*2/*c*),^24^ β-Zn_2_V_2_O_7_ (S. G. *C*2/*m*, isomorphous to Cd_2_V_2_O_7_),^25^ γ-Zn_2_V_2_O_7_ (S. G. *P*1̄, isomorphous to Mg_2_V_2_O_7_),^26^ δ-Zn_2_V_2_O_7_ (S. G. *Pnma*, isomorphous to Hg_2_V_2_O_7_),^27^ ε-Zn_2_V_2_O_7_ (S. G. *P*2_1_/*c*, isomorphous to Pb_2_V_2_O_7_),^28^ ω-Zn_2_V_2_O_7_ (S. G. *P*1̄, isomorphous to Sr_2_V_2_O_7_),^29^ κ-Zn_2_V_2_O_7_ (S. G. *P*2_1_/*c*, isomorphous to Ni_2_V_2_O_7_).^30^ From now on, we will use the names α, β, γ, δ, ε, ω and κ for denoting the different phases in the rest of the manuscript.

The diagonalization of the Fock matrix has been performed at adequate *k*-point grids in the reciprocal space which depend on the phase under treatment using Pack–Monkhorst/Gilat shrinking factors (IS = ISP = 4), being the total number of *k*-points 24, 24, 36, 27, 30, 36 and 36 in the α, β, γ, δ, ε, ω and κ structures, respectively. The number of atoms in the unit cells is 22, 11, 22, 44, 44, 44 and 44 for the α, β, γ, δ, ε, ω, and κ structures, respectively. Thresholds controlling the accuracy of the calculation of Coulomb and exchange integrals have been set to 10^−8^ and 10^−14^ which assure a convergence in total energy better than 10^−7^ hartree in all cases, whereas the percent of Fock/Kohn–Sham matrices mixing has been set to 40 (IPMIX = 40). Since the choice of the exchange–correlation functional is of critical importance as it has a significant influence on the properties obtained,^31^ a complete structure optimization in terms of unit-cell parameters and atomic positions of the different Zn_2_V_2_O_7_ structures has been performed by using the B3LYP, HSE06, and PBE functionals.

In a previous study on ZnV_2_O_6_ (zinc metavanadate)^9^ it was shown that the empirical-correction scheme to energy that considers the long-range dispersion contributions proposed by Grimme^32^ should be included for properly describing this zinc vanadate. However, in the case of Zn_2_V_2_O_7_ (zinc pyrovanadate) we have found that structural and electronic properties were practically unaltered when including the Grimme dispersion correction. In particular, in α-Zn_2_V_2_O_7_, the unit-cell parameters at ambient pressure change 1% when including this semi-empirical correction. In addition, the shape of the electronic band structure and value on the band-gap energy remain nearly unchanged when including the Grimme correction. This indicates that long-range correlation interactions which capture van der Waals forces are weak in Zn_2_V_2_O_7_ not playing, as first-approximation, any relevant role in Zn_2_V_2_O_7_. Consequently, for the sake of computational efficiency, the Grimme extension was not included in the HP calculations.

In order to determine the thermodynamically stable phases and the possible phase transitions, the total energy was calculated as a function of the unit-cell volume through the optimization of the crystal structure. From total-energy calculations, the pressure (*P*)–volume (*V*) relationship was obtained from the energy (*E*) *versus* volume (*V*) curves by means of an equation of state (EOS) fit using a third-order Birch–Murnaghan EOS;^33^ where the fitting parameters were the volume at zero pressure (*V*_0_), the zero pressure bulk modulus (*B*_0_), and its pressure derivative (
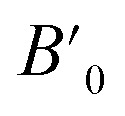
). Then, the enthalpy (*H* = *E* + *P* × *V*) of different polymorphs was determined as a function of pressure. The enthalpy/pressure curves obtained for Zn_2_V_2_O_7_ polymorphs were used to probe the thermodynamic stability of the system and to determine the values of theoretical transition pressures. The electronic-density of states (DOS) and band structure have been calculated for different polymorphs based on the optimized geometries. They allow determining the pressure dependence of the band-gap energy.

## 3. Results and discussion

### 3.1 Pressure-induced phase transitions

**Fig. 1:**
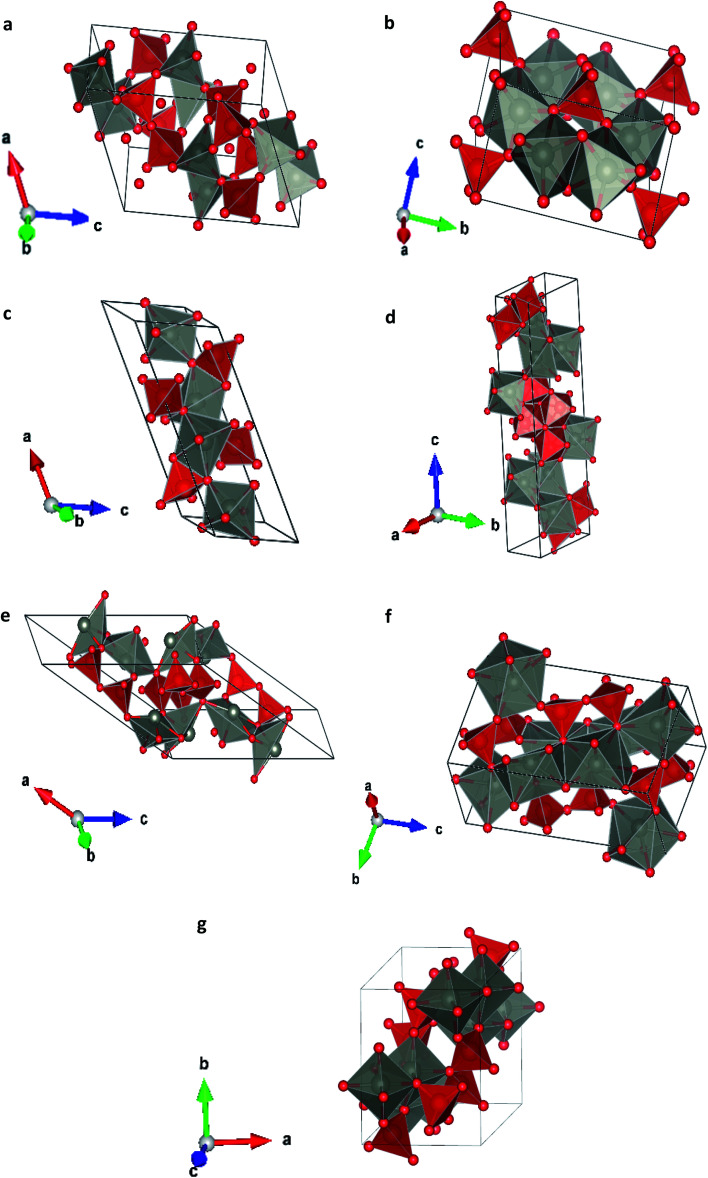
Different crystallographic structures proposed, (a) α-phase, (b) β-phase, (c) γ-phase, (d) δ-phase, (e) ε-epsilon, (f) ω-phase, (g) κ-phase.

In order to study the effect of pressure in the crystal structure we have performed calculations considering seven different potential polymorphs (described explicitly in the previous section). These structures include the three phases that were previously found in experiments (α, β, and γ),^7^ which were already mentioned in the introduction, and four additional phases. These four phases have crystal structures isomorphic to other pyrovanadates. They were considered in our study because according to crystal chemistry arguments they are susceptible to become stable under HP conditions in Zn_2_V_2_O_7_.^24^ Schematic representations of the crystal structures of the seven phases are shown in Fig. 1.

We have performed calculations using three different functionals and found that according to all of them that at ambient pressure α-Zn_2_V_2_O_7_ is the minimum enthalpy phase. We also found that the B3LYP functional is the one that best describe the crystal structure of Zn_2_V_2_O_7_ at ambient conditions. Values of the obtained unit-cell parameters are summarized in Table 1. There it can be seen that B3LYP underestimates the unit-cell volume by 1.7%. In contrast, the HSE06 and PBE functionals underestimate the volume by 4.4% and 2.8%, respectively (see Table 1). In Table 1 it can be also seen that previous calculations using the PBE formulation within the CASTEP code largely underestimated the unit-cell volume of Zn_2_V_2_O_7_. The atomic positions here calculated are given in Table 2, where it can be seen an excellent agreement of present calculations using the B3LYP functional with the experiments.^25^

Unit-cell parameters and volume (per formula unit) of different Zn_2_V_2_O_7_ structures calculated with three different functionals (a) α, (b) β, (c) γ, (d) δ, and (e) κ. They are compared with experimental and calculated unit-cell parameters reported in the literature^7,24^

**Table:** 

(a)	B3LYP	HSE06	PBE	Exp.^24^	CASTEP^2^
*a* (Å)	7.3111	7.2008	7.2372	7.429(5)	7.156(0.027)
*b* (Å)	8.2665	8.2053	8.2333	8.340(3)	7.903(0.022)
*c* (Å)	10.1095	10.0320	10.1286	10.098(3)	10.167(0.028)
*β* (°)	110.91	110.52	110.69	111.37(5)	111.37
*V*_0_ (Å^3^)	142.7	138.8	141.2	145.7(1)	133.9

**Table:** 

(b)	B3LYP	HSE06	PBE	Exp.^7^
*a* (Å)	6.7274	6.4925	6.5135	6.648(5)
*b* (Å)	8.3913	8.3992	8.4469	8.446(6)
*c* (Å)	4.9888	4.9423	4.9973	4.9606(5)
*β* (°)	105.84	103.57	103.54	106.0(2)°
*V*_0_ (Å^3^)	135.5	131.0	133.7	133.9(3)

**Table:** 

(c)	B3LYP	HSE06	PBE
*a* (Å)	14.1808	13.3671	13.5985
*b* (Å)	5.4681	5.4266	5.4417
*c* (Å)	5.1222	5.0044	5.0977
*α* (°)	76.78	79.97	78.41
*β* (°)	110.56	106.54	107.92
*γ* (°)	130.86	130.19	130.42
*V*_0_ (Å^3^)	140.6	132.9	136.6

**Table:** 

(d)	B3LYP	HSE06	PBE
*a* (Å)	6.9068	6.8402	6.8648
*b* (Å)	3.5667	3.5460	3.5779
*c* (Å)	19.6966	19.4272	19.6090
*V*_0_ (Å^3^)	121.3	117.8	120.4

**Table:** 

(e)	B3LYP	HSE06	PBE
*a* (Å)	6.6247	6.5664	6.6145
*b* (Å)	8.4115	8.3480	8.3944
*c* (Å)	9.5001	9.4223	9.4919
*β* (°)	100.47	100.39	100.29
*V*_0_ (Å^3^)	130.1	127.0	129.6

**Table:** Atomic positions in the crystallographic cell of α-phase. The columns in the left are the theoretical calculations with B3LYP functional and those for the right are of the bibliography^24^

Atom	Site	*x*	*y*	*z*	*x*	*y*	*z*
Zn	8f	0.4551	0.1797	0.0202	0.45	0.1760	0.0196
V	8f	0.2096	0.0011	0.2066	0.202	0.0049	0.206
O_1_	4e	0	0.0412	0.25	0	0.0612	0.25
O_2_	8f	0.0910	0.4782	0.1401	0.102	0.481	0.138
O_3_	8f	0.2458	0.1606	0.1104	0.244	0.154	0.106
O_4_	8f	0.3311	0.3336	0.3900	0.347	0.335	0.386

**Fig. 2:**
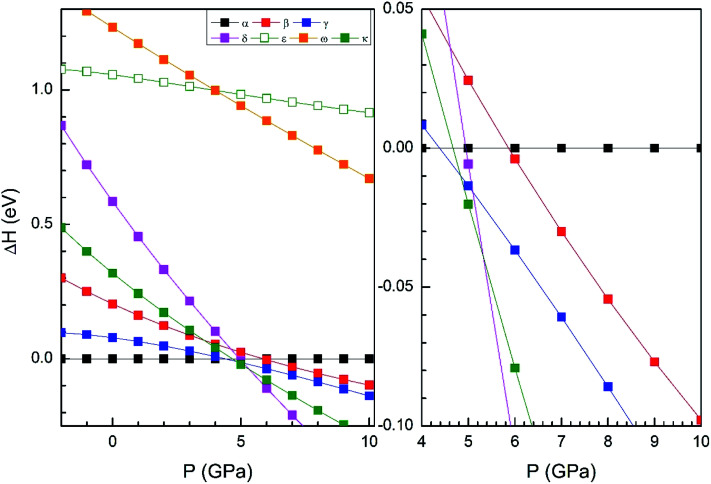
Relative enthalpy to alpha phase dependence of the pressure of different crystal structures with the functional B3LYP. In the center we have expanded the region where the phase transition occurs.

From our simulations, we have found that the three functionals we used in the calculations give a qualitatively similar HP structural sequence. Thus, to avoid redundancies we will mainly focus on describing the results obtained using B3YLP, the approach that better describe the ambient-pressure structure. In order to determine the thermodynamically most-stable phase of Zn_2_V_2_O_7_ at different pressures (determining therefore possible phase transitions) we have represented the enthalpy *versus* pressures for different phases in Fig. 2 (results calculated using B3YLP). This figure shows that the α-phase is the lowest enthalpy phase at ambient pressure and therefore it is the most stable, in agreement with experiments.^7^ We observe that the B3LYP functional predicts the first transition at 4.4 GPa approximately (HSE06 finds it at 2 GPa and PBE at 2.1 GPa). The α-phase is the one with lowest enthalpy (*i.e.* the most stable phase) up to this pressure, becoming the γ-phase the lowest enthalpy phase beyond 4.4 GPa, which supports the occurrence of the α–γ transition at this pressure. Calculations also predict a second phase transition at 4.8 GPa from the γ phase to the κ-phase followed by a subsequent transition to the δ-phase at 5.3 GPa. This phase is the lowest enthalpy phase among the seven considered phases up to 10 GPa.

We will compare now our finding with experiments.^7^ Experiments found first a α–β transition, being the onset of the transition at 0.7 GPa, but coexisting both phases up to 1.1 GPa. Experiments also found the β–γ transition at 3.8 GPa. Our calculations, in contrast, predict a direct α–γ transition at 4.4 GPa. Thus both experiments and calculations found the γ-phase to be the stable polymorph after a compression of around 4 GPa. We will comment next on the fact that the β-phase is not find by calculations between the α- and γ-phases. In Fig. 2 it can be seen that the β phase is competitive in enthalpy to the γ-phase (the difference is smaller than 0.05 eV). Thus, the experimental finding of the β-phase^7^ as the first HP phase of Zn_2_V_2_O_7_ could be related to the existence of a kinetic barrier blocking the transition to the γ-phase, which is a typical phenomenon of complex oxides under compression.^34,35^ The fact that the β-phase has been also obtained at high-temperature and ambient conditions^36^ supports the kinetic barrier hypothesis. The same argument applies for other phases with lower enthalpy than the β-phase at the theoretical transition pressure (for instance the κ-phase). Another possibility, is that entropy effects related to temperature (calculations have been carried out at 0 K) could favor the transition to the β-phase at room temperature.^37^ Performing finite temperature calculations is beyond the scope of the present study. Regarding the other candidate structures, from Fig. 2 it can be concluded that the ε and ω phases can be ruled out as possible candidates for HP polymorphs of Zn_2_V_2_O_7_. On the other hand, the finding of a post-γ-phase in the experiments is consistent with our finding that the δ- and κ-phases are more stable than the α-phase beyond 5.3 GPa.

In Table 1 we report the calculated unit-cell parameters for the β-phase. They agree well with those determined from HP XRD experiments.^5^ The calculated atomic positions (given in Table 3) also agree well with the literature.^36^ The similitude between the calculated and measured crystal structure of the β-phase supports the α–β transition found in experiments. In addition, the similarities of the calculated γ-phase with the γ-phase found in the experiments, support the observation of this phase at 3.8 GPa.

**Table:** Atomic positions in the crystallographic cell of β-phase. The columns in the left are the theoretical calculations with B3LYP functional and those in the right are of the bibliography^25^

Atom	Site	*x*	*y*	*z*	*x*	*y*	*z*
Zn	4h	0	0.3121	0	0	0.3158	0
V	4i	0.2327	0	0.4061	0.2174	0	0.4049
O_1_	2a	0	0	0.5	0	0	0.5
O_2_	8j	0.2248	0.1677	0.2162	0.2151	0.1519	0.2085
O_3_	4i	0.5790	0	0.2775	0.5909	0	0.2950

**Fig. 3:**
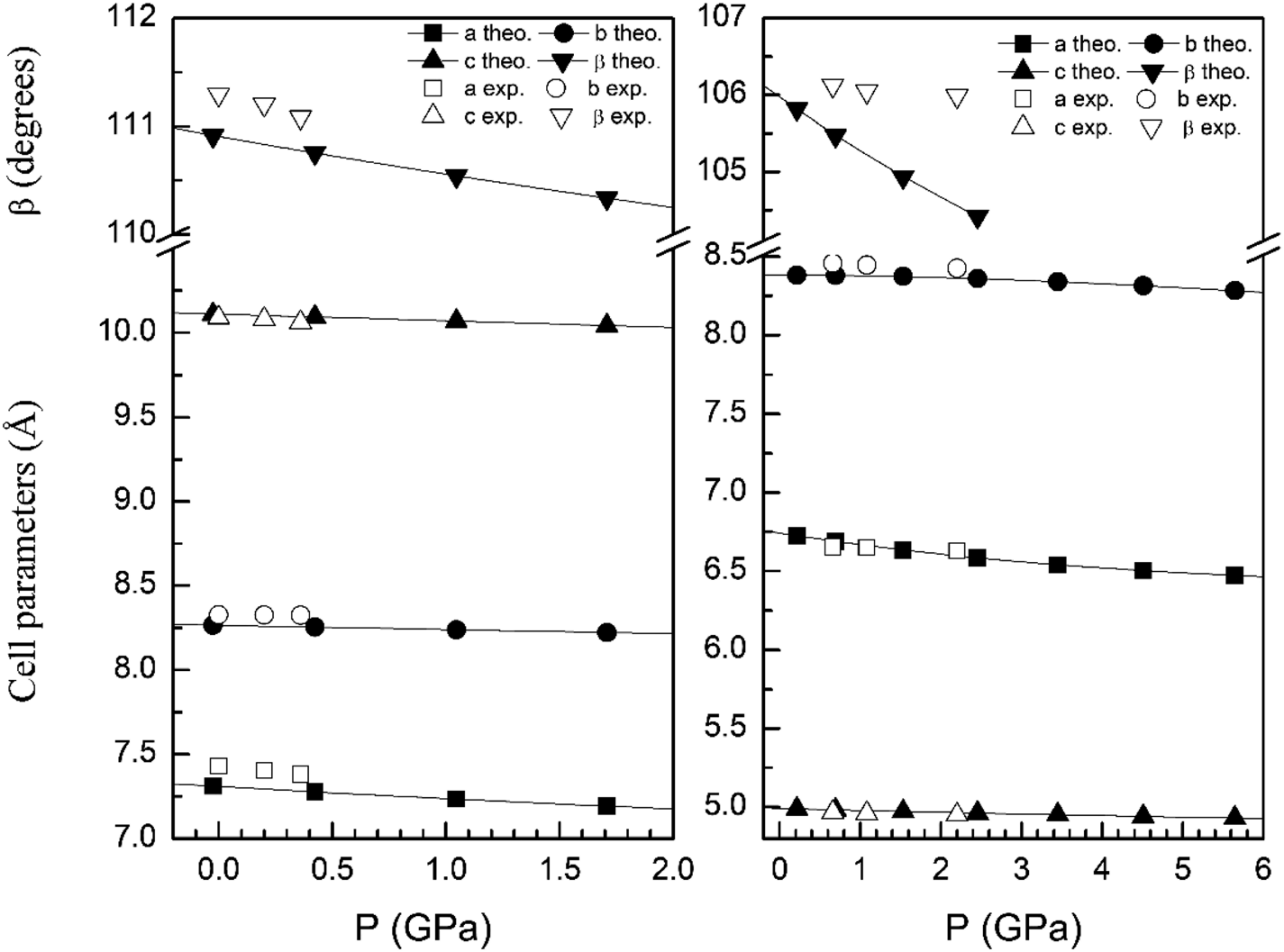
Cell parameters of α- (left graph) and β- (right graph) phases *versus* the pressure, compared with experimental results.^7^

In order to display further evidence that the functional B3LYP describes well the structure of the α and β phases, and the changes induced by pressure on it, we compare the calculated and measured pressure dependence of unit-cell parameters. Fig. 3 shows the unit-cell parameters of α and β phases *versus* the pressure. The results from calculations agree well with experiments^5^ (maximum 2% of relative error).

**Fig. 4:**
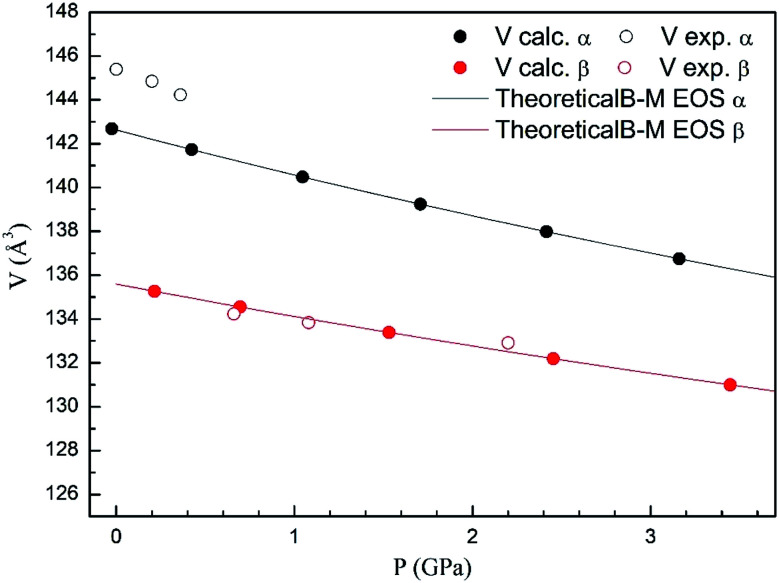
Unit cell volume of α- and β-phases *versus* the pressure, compared with experimental results.^7^

In Fig. 4 we compare the pressure dependence of the volume showing a good agreement with experiments.^7^ A 4% collapse of the volume happening at the α–β transition. For the γ-phase, no comparison can be done for the pressure dependence of unit-cell parameters with experiments because this experimental information is not available yet. According to the present calculations, in the pressure range of stability of the γ-phase, it has a similar volume and compressibility than the β-phase.

For completeness and favoring comparison with future studies, in Tables 4–6 we report the calculated atomic positions for the γ, κ- and δ-phases, respectively. The atomic positions of the γ-phase were not determined from previous powder XRD experiments, and the crystal structure of the post-γ-phase of Zn_2_V_2_O_7_ has not been even proposed yet. Thus, the results of the Tables 3–5 cannot be compared with previous studies, but they can help as a guide for the solution of the crystal structures of the γ-phase and post-γ-phase of Zn_2_V_2_O_7_ in future studies. According to our calculations, the post-γ-phase could be either the κ- or δ-phase, but as will comment below, we predict the κ-phase to be most probably the post-γ-phase.

**Table:** Calculated atomic positions in the crystallographic cell of γ-phase

Atom	Site	*x*	*y*	*z*
Zn_1_	2i	0.1151	0.3706	0.4627
Zn_2_	2i	0.4229	0.6311	0.4582
V_1_	2i	0.6344	0.9688	0.0776
V_2_	2i	0.8524	0.9714	0.9077
O_1_	2i	0.7300	0.9064	0.9990
O_2_	2i	0.9564	0.9803	0.2178
O_3_	2i	0.7329	0.3200	0.2371
O_4_	2i	0.9203	0.3175	0.7352
O_5_	2i	0.4659	0.0292	0.2283
O_6_	2i	0.2278	0.3472	0.3157
O_7_	2i	0.4553	0.3413	0.7158

**Table:** Calculated atomic positions of the κ-phase

Atom	Site	*x*	*y*	*z*
Zn_1_	4e	0.1467	0.1232	0.4640
Zn_2_	4e	0.3107	0.3921	0.6823
V_1_	4e	0.3598	0.7585	0.5307
V_2_	4e	0.1921	0.0198	0.8157
O_1_	4e	0.6102	0.1285	0.1212
O_2_	4e	0.4312	0.1288	0.3984
O_3_	4e	0.1687	0.3712	0.4605
O_4_	4e	0.2573	0.3621	0.1862
O_5_	4e	0.6823	0.3691	0.3476
O_6_	4e	0.0250	0.0831	0.2462
O_7_	4e	0.8504	0.3770	0.0082

**Table:** Calculated atomic positions of the δ-phase

Atom	Site	*x*	*y*	*z*
Zn_1_	4c	0.3094	0.25	0.7560
Zn_2_	4c	0.0684	0.25	0.3500
V_1_	4c	0.0422	0.25	0.1294
V_2_	4c	0.2887	0.25	0.5143
O_1_	4c	0.4311	0.25	0.7985
O_2_	4c	0.3869	0.25	0.3048
O_3_	4c	0.2606	0.25	0.1701
O_4_	4c	0.1240	0.25	0.0281
O_5_	4c	0.4569	0.25	0.6058
O_6_	4c	0.0832	0.25	0.5517
O_7_	4c	0.2507	0.25	0.4289

### 3.2 Bulk modulus and compressibility tensor

**Fig. 5:**
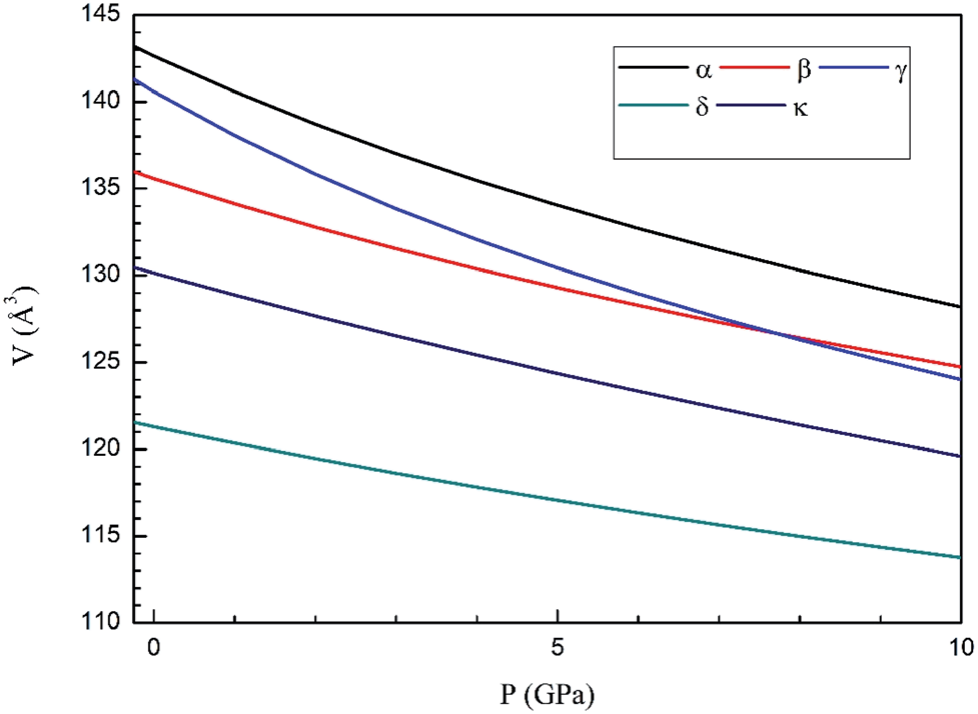
Pressure dependence of the unit-cell volume of Zn_2_V_2_O_7_ in B3LYP of α-, β-, γ-, δ- and κ-phases using A Birch–Murnaghan equation of state.

Next we will discuss the volume compressibility of the different phases. In Fig. 5 we report the pressure dependence of the unit-cell volume of the five relevant phases for this study up to 10 GPa. The dependence of the volume with pressure is well described with a third-order Birch–Murnaghan equation of state (EOS).^33^ In Table 7 we report the volume at zero pressure (*V*_0_), bulk modulus (*B*_0_), and its pressure derivative (
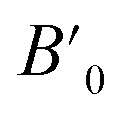
) obtained for the EOS of different phases. Results obtained from different functionals are included in the table. All functionals give a qualitative similar behavior. The β-phase is less compressible than the α- and γ-phases, and the δ- and κ-phases are the least compressible phases. The bulk modulus of the different phase correlates with the inverse of the unit-cell volume at ambient pressure. The larger the volume the more compressible the phase, as expected.^38^ From Fig. 5 it can be seen that the transition from the γ-phase to the κ- and δ-phase involve a large volume collapse. In the first case the change in volume is nearly 6% (similar to the volume change between α and β or γ), but in the second case, the volume collapse is more than 13%, which is very large. The large volume change that occurs from γ to δ also supports the idea that a kinetic barrier could block the γ–δ transition, being more easily to obtain experimentally the transition for the γ-phase to a metastable κ-phase, which has associated a smaller volume collapse and consequently has associated less drastic changes in bonds.

**Table:** The unit-cell volume (Å^3^), bulk modulus (GPa) and bulk modulus pressure derivative at ambient pressure determined using a third-order Birch–Murnaghan EOS

Phase	B3LYP			HSE06			PBE		
	*V*_0_	*B*_0_	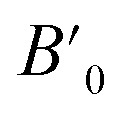	*V*_0_	*B*_0_	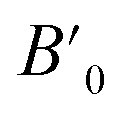	*V*_0_	*B*_0_	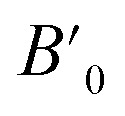
α	142.63	65.7	6.3	138.73	59.6	1.1	140.92	57.0	0.6
β	135.59	87.8	7.7	131.09	115.4	5.4	133.71	113.9	3.6
γ	140.57	52.5	6.3	133.68	78.6	7.7	136.77	70.6	5.4
δ	121.29	124.9	6.7	117.83	135.4	6.3	120.43	126.6	5.9
κ	130.14	101.2	3.7	127.02	101.0	3.7	129.70	89.2	4.2

**Fig. 6:**
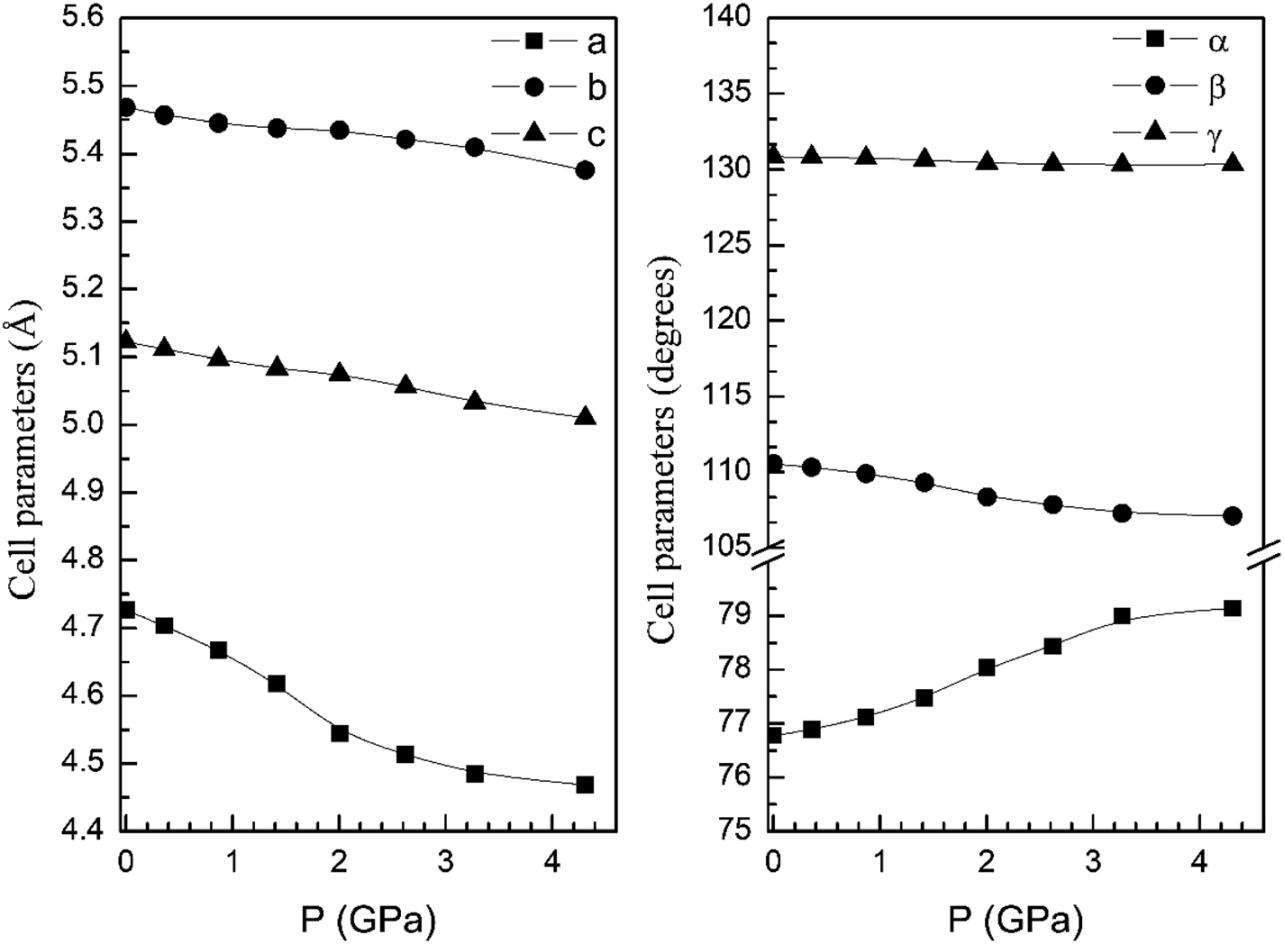
Unit-cell parameters of γ-phase *versus* the pressure.

**Fig. 7:**
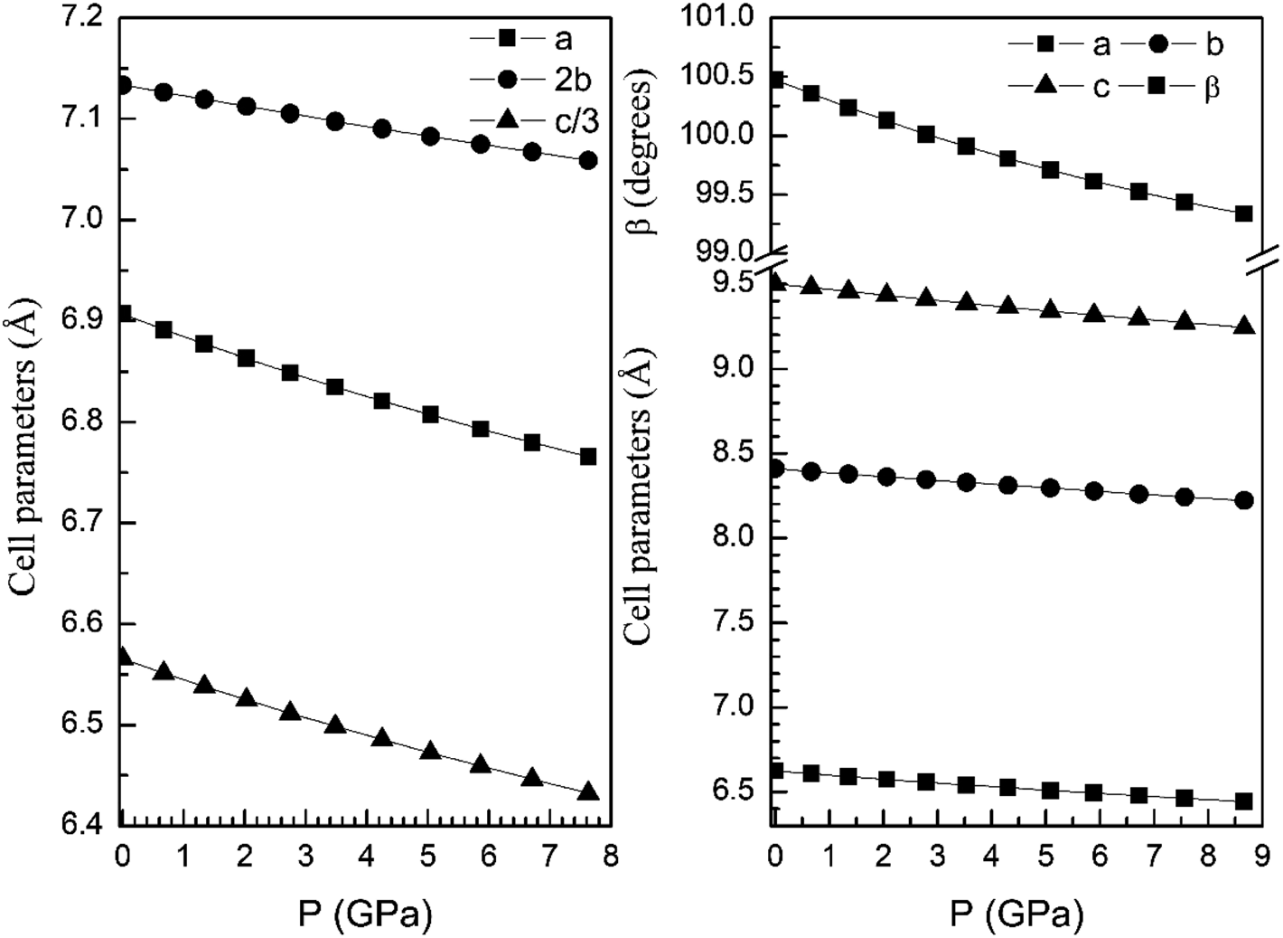
Unit-cell parameters of δ- (left graph) and κ-phases (right graph) *versus* the pressure. For the δ-phase we plot *c*/3 and 2*b* to facilitate the comparison between unit-cell parameters.

From our calculations we have also found that the response to pressure of the different polymorphs is anisotropic. This can be clearly seen in Fig. 3 for the α- and β-phases. A similar qualitative behavior has been found for the other polymorphs. This can be seen in Fig. 6 and 7 where we represent the pressure dependence of the unit-cell parameters for the γ-, δ-, and κ-phases. For instance, in the δ-phase the less compressible axis is the *b*-axis (see Fig. 7); being the linear compressibilities *κ*_*a*_ = *κ*_*c*_ = 3.26 × 10^−3^ GPa^−1^ and *κ*_*b*_ = 1.63 × 10^−3^ GPa^−1^. From these values a bulk modulus of 122 GPa is obtained in very good agreement with our EOS calculations (see Table 7). In the other structures is not so evident to determine the most compressible direction, given their monoclinic or triclinic symmetry. For the triclinic structure, the behavior is non-isotropic but also strongly non-linear as can be seen in Fig. 6. In the case of the monoclinic structures, the compressibility is described by a symmetric tensor with four elements different than zero.^39^ We have calculated it for the α-, β-, and γ-phases (the three monoclinic polymorphs) at zero pressure to further investigate its behavior under compression. The eigenvalues and eigenvectors of the compressibility tensor describe the magnitudes and directions of the principal axes of compression.^40^ We have obtained them for α-Zn_2_V_2_O_7_, β-Zn_2_V_2_O_7,_ and γ-Zn_2_V_2_O_7_ using PASCAL.^41^ Their values are given in Table 8.

**Table:** Eigenvalues, *λ*_*i*_, and eigenvectors, *e*_ν*i*_, of the isothermal compressibility tensor of α-phase (top), β-phase (center), and κ-phase (bottom) at 0 GPa

*λ*_1_ = 8.4(1) × 10^−3^ GPa^−1^	*e*_ν1_ = (0.9753, 0, −0.2208)
*λ*_2_ = 1.4(1) × 10^−3^ GPa^−1^	*e*_ν2_ = (0, −1, 0)
*λ*_3_ = 1.0(2) × 10^−3^ GPa^−1^	*e*_ν3_ = (−0.6404, 0, −0.7680)
*λ*_1_ = 5.5(3) × 10^−3^ GPa^−1^	*e*_ν1_ = (0.8410, 0, −0.5410)
*λ*_2_ = 3.3(1) × 10^−3^ GPa^−1^	*e*_ν2_ = (0, −1, 0)
*λ*_3_ = −0.9(1) × 10^−3^ GPa^−1^	*e*_ν3_ = (−0.4422, 0, −0.8969)
*λ*_1_ = 4.04(4) × 10^−3^ GPa^−1^	*e*_ν1_ = (−0.8321, 0, 0.5546)
*λ*_2_ = 2.59(1) × 10^−3^ GPa^−1^	*e*_ν2_ = (0, −1, 0)
*λ*_3_ = 1.84(1) × 10^−3^ GPa^−1^	*e*_ν3_ = (0.8117, 0, 0.5840)

We have found that in the α-phase, the major compression direction is approximately parallel to the [501̄] crystallographic axis, being this direction at least six times more compressible than any other direction. The minimum compression direction is also in the plane perpendicular to the unique *b*-axis, approximately along the [405] crystallographic axis, but the compressibility in this direction is similar to the compressibility along the *b*-axis (see Table 8). From PASCAL we have obtained a bulk modulus of 62.3(5) GPa, which is in agreement with the result obtained from our EOS analysis.

In the case of the β-phase, we found that upon compression two principal axes undergoes contraction but the third axis undergo an expansion, as can be seen by the negative value of the eigenvalue in Table 8. This anomalous behavior of lattice directions under pressure suggests an auxetic behavior of β-Zn_2_V_2_O_7_.^42^ This conclusion should be supported by future elastic constants calculations (which are beyond the scope of this work) and a detail evaluation of Poisson ratios.^43,44^ As can be seen in Table 8, in the β-phase, the direction the maximum compression is nearly parallel to the [302̄] crystallographic axis, being the linear compressibility along this direction 5/3 times the one along the *b*-axis. On the other hand, the direction that slightly expands under compression is nearly parallel to the [102] crystallographic axis. From PASCAL^41^ we have obtained a bulk modulus of 89.2(5) GPa, which is in agreement with the result obtained from our EOS analysis.

In the case of the κ-phase, the direction the maximum compression is nearly parallel to the [3̄02] crystallographic axis, being the linear compressibility along this direction more than two times than the minimum compressibility (see Table 8). On the other hand, the direction of minimum compressibility is nearly parallel to the [403] crystallographic axis. From PASCAL^41^ we have obtained a bulk modulus of 101.6(5) GPa, which is in agreement with the result obtained from our EOS analysis.

Additional information on the structural changes induced by pressure can be obtained by the analysis of coordination polyhedra. In the Table 9 we report the calculated average bond distances (Zn–O and V–O) of the different phases at ambient pressure. We also include other parameters relevant for the polyhedra like the distortion index and coordination number (CN). There it can be seen that the transition from α to β involves little changes in the coordination polyhedra. In fact, the transition can be related to a tilting of ZnO_5_ polyhdra of the α-phase, which favour formation of ZnO_6_ distorted octahedra in the β-phase, with four short equatorial bonds (∼2.05 Å) and two long apical distances (∼2.4 Å). Thus the effective coordination number (as defined by Hoppe *et al.*^45^) of ZnO_6_ in the β-phase is smaller than five (4.92), as in the α-phase. Thus the above mentioned transition only requires small collective displacements of atoms, being probably a displacive transition. In contrast, all the rest of the HP phases involve an abrupt increase of the coordination number (CN) of Zn atoms, which have a truly octahedral coordination in the γ- δ-, and κ-phases, with an effective CN larger than five and in cases close to six. Such a change in the sphere of coordination of Zn will imply a very important structural reorganization, being probably the transition reconstructive. This observation supports the existence of a kinetic barrier for the proposed reconstructive α–γ transition, blocking this transition and allowing the occurrence of the displacive α–β transition. Another important fact to highlight is that the Zn coordination in the β-phase gradually increases under compression, reaching a value of 5.3 a 4 GPa, which is a coordination number closer to that of Zn in the γ-phase. Therefore, the β-phase probably plays the role of a bridge metastable phase between α and γ, which is fully consistent with the kinetic barrier argument.

**Table:** Average of the bond-length of different polyhedra for each phase, along with the distortion and effective coordination number (CN)

Sample	Zn–O	Distortion	CN	V–O	Distortion	CN
α	2.0327	0.0216	4.89	1.7161	0.0204	3.90
β	2.1924	0.0675	4.92	1.6334	0.0140	3.97
γ	2.0856	0.0229	5.79	1.6998	0.0328	3.81
δ	2.4225	0.0620	5.22	1.9212	0.0738	4.51
κ	2.0556	0.0131	5.96	1.7350	0.0313	3.83

If we look to the V coordination polyhedron, in Table 6 it can be seen that most structures have V atoms in the tetrahedral coordination as in the α-phase. Only the δ-phase shows a change in the V polyhedra. In this structure V is in octahedral coordination, being the effective CN 4.5. Such a change in the V coordination is quite unusual at pressure below 10 GPa (ref. 8) and could involve quite large kinetic barriers, requesting therefore the phase transition the simultaneous application of pressure and temperature. On the other hand, by compression at room temperature, more probably the γ–κ transition will be observed instead of the γ–δ transition. Future experiments should be performed to test these predictions.

### 3.3 Electronic properties

**Fig. 8:**
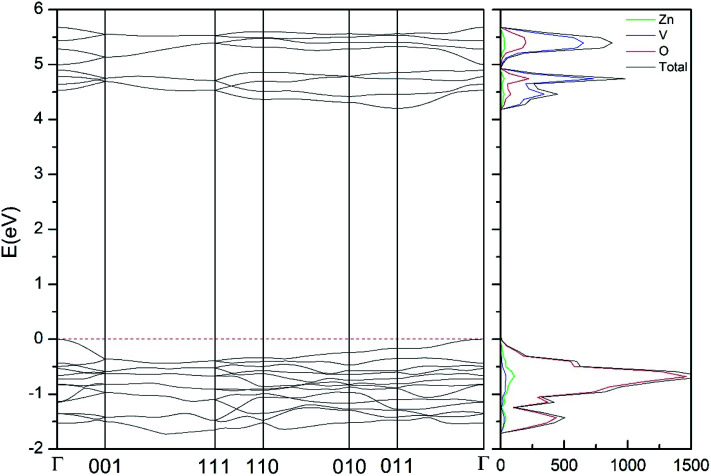
Band structure and DOS of the α-phase calculated at ambient pressure with the B3LYP potential.

**Fig. 9:**
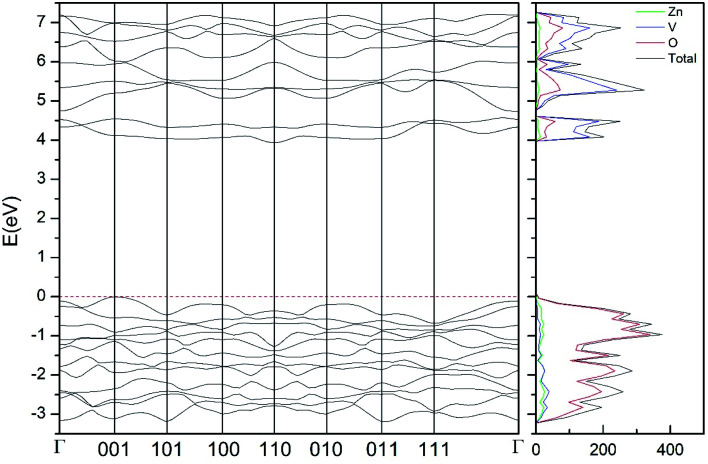
Band structure and DOS of the β-phase calculated at ambient pressure with the B3LYP potential.

**Fig. 10:**
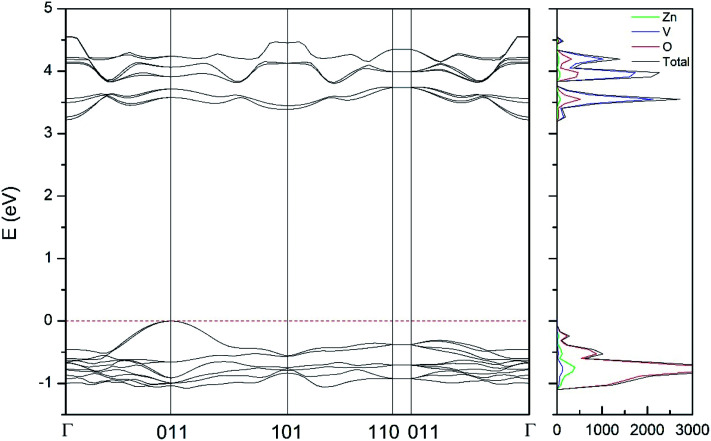
Band structure and DOS of the δ-phase calculated at ambient pressure with the B3LYP potential.

**Fig. 11:**
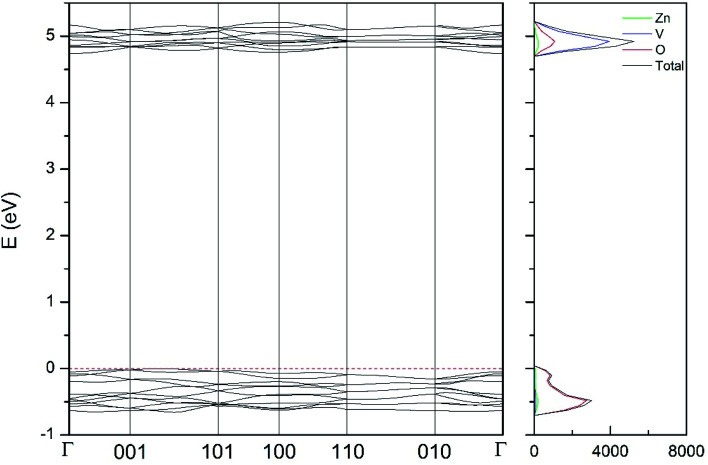
Band structure and DOS of the κ-phase calculated at ambient pressure with the B3LYP potential.

**Fig. 12:**
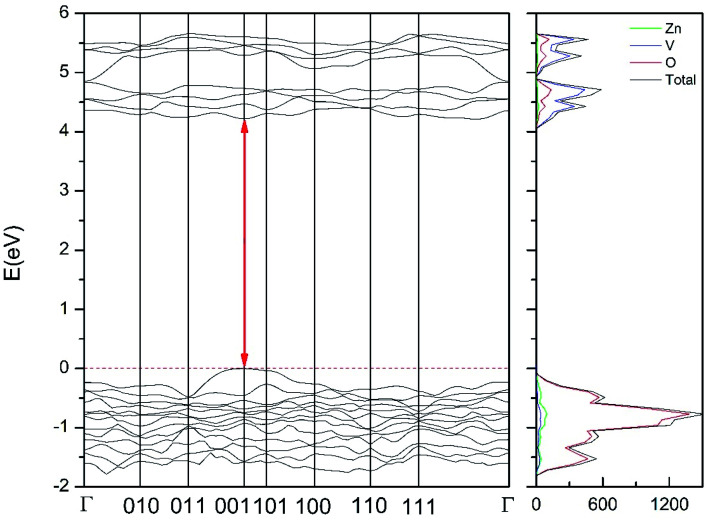
Band structure and DOS of the γ-phase calculated at ambient pressure with the B3LYP potential. The red line with arrows shows the direct band gap.

In addition to the structural study, we have explored the influence of pressure in the electronic properties of Zn_2_V_2_O_7_. The calculate band structure and electronic DOS for the different phases are shown in Fig. 8–12. We have found that at ambient pressure Zn_2_V_2_O_7_ is an indirect gap material with a wide band gap. The calculated value of the band-gap energy (*E*_g_) at ambient pressure using B3LYP is 4.2 eV. Using HSE06 we obtained 3.98 eV and using PBE we obtained 2.29 eV. The B3LYP and HSE06 values are slightly larger than the experimental value determined from photoluminescence (3.5 eV).^16^ This ultraviolet band-gap of Zn_2_V_2_O_7_ has been challenged by diffuse-reflectance measurements^14^ and calculations.^13–15^ Previous calculations have estimated the band-gap energy of the α-phase to be 2.5 eV and reflectance experiments report a band gap 2.86 eV.^13–15^ However, the are several facts that points towards a band-gap underestimation in reflectance measurements and previous calculations. A first fact is that the reflectance measurements were performed in doped samples which might induce a sub-bandgap optical absorption.^46^ A second fact that reflectance measurements only give a lower limit for *E*_g_ (ref. 46) and not to the fundamental band-gap. On the other hand, previous calculations have been carried out using the PBE functional, which is known to underestimate the band gap of vanadates.^47^ These reasons and the present calculations indicate that the most correct determination of the band-gap is 3.5 eV. This is supported not only our calculations but also by the white color of Zn_2_V_2_O_7_. In Table 10 we report the calculated *E*_g_ for different phases. There it can be seen that the PBE functional considerably underestimate *E*_g_, as expected,^40,47^ while HSE06 functional provides *E*_g_ values intermediate between B3LYP and PBE formulations, which only deviate by 10% from the experimental value. The fact that PBE underestimate the band-gap energy by approximately 1 eV (in our and previous studies^13^) is consistent with recent studies from related vanadates,^47^ which showed that the Hubbard U contribution should be included in PBE calculations to properly describe electronic structures of multiple vanadates.

**Table:** Band-gap energy (*E*_g_) of the different phases calculated with DFT calculations and different potentials. The pressure coefficient at ambient pressure (d*E*_g_/d*P*) and the band-gap Grüneisen parameter (*γ*_gap_) obtained from B3LYP calculations are also included. The position of the top of the conduction band and bottom of the valence band are also indicated

Phase	Gap	Bottom	Top	B3LYP			HSE06 *E*_g_ (eV)	PBE *E*_g_ (eV)
				*E*_g_ (eV)	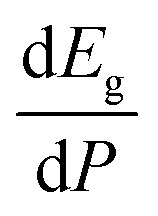 (meV GPa^−1^)	*γ*_gap_		
α	Indirect	Γ	011	4.20	−32	−0.45	3.98	2.29
β	Indirect	001	110	3.95	−31	−0.91	3.63	2.00
γ	Direct	001	001	4.17	−65	−1.22	3.78	2.13
δ	Indirect	011	Γ	3.22	−26	−1.09	2.91	1.46
κ	Indirect	001	Γ	4.74	6.1	0.16	4.56	2.80

As can be seen in Fig. 8, we have found that α-Zn_2_V_2_O_7_ has an indirect band gap. The position of the maximum of the valence band (VB) and the minimum of the conduction band (CB) are given in Table 10. We have also found that most relevant phases are indirect materials and have a large band gap (see Table 10 and Fig. 8–12). The only exception is γ-Zn_2_V_2_O_7_, which is a wide gap material but with a direct band gap at ambient pressure. From the calculated partial electronic DOS we have found that the states at the top of the VB are dominated by O 2p orbitals, while the states near the bottom of the CB are dominated by V 3d orbitals, which are partially hybridized with O 2p orbitals. The Zn states have a negligible contribution to the states near the Fermi level. This feature is common to α-, β-, γ-, δ-, and κ-phases. It is also similar to the topology of the band structure of ZnV_2_O_6_.^7^ In fact, it is not surprising that the different polymorphs of zinc vanadates have energy band gap in the 3–4 eV region, and that this value is comparable to the band-gap energy of multiple orthovanadates.^48^ This is not a mere coincidence, but a consequence of the fact than Zn orbitals (or those from equivalent cations) have little influence in the states near the Fermi level, being them dominated by V and O orbitals as described above.

**Fig. 13:**
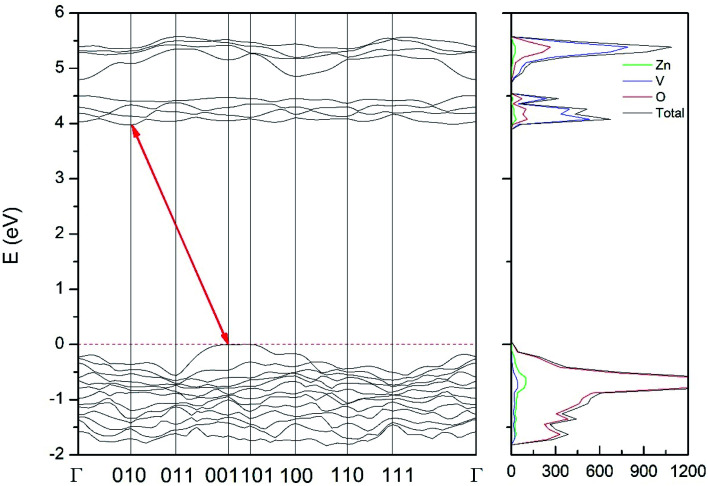
Band structure and DOS of the γ-phase calculated at 4 GPa with the B3LYP potential. The red line with arrows shows the indirect band gap.

Regarding the influence of pressure in band structure of different polymorph, we have found that in most polymorphs pressure modifies the band-gap energy but does not change the topology of the band structure. Only in the case of γ-Zn_2_V_2_O_7_ we found that pressure induces a band crossing, changing at 1.5 GPa the nature of the bang gap from direct (top of the valence band and bottom of the conduction band at 001) to indirect (010 becomes the absolute minimum of the conduction band at 1.5 GPa). To illustrate this fact, we show in Fig. 13 the calculated band structure at 4 GPa of γ-Zn_2_V_2_O_7_, which can be compared with its band structure at ambient pressure, shown in Fig. 12. The distortion of the topology of the band structure of triclinic γ-Zn_2_V_2_O_7_ and the observed band crossing could be probably related to the non-linear behavior of unit-cell parameters in this polymorph that we previously described (see Fig. 6).

**Fig. 14:**
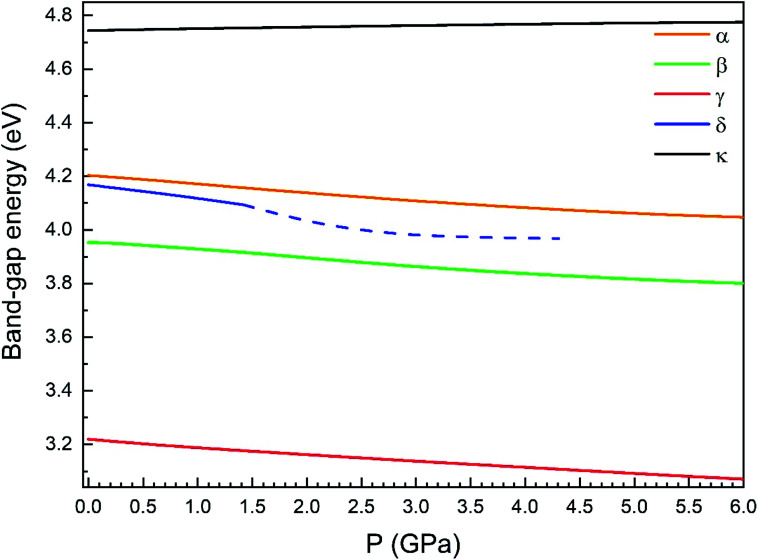
Evolution of the band-gap energy with pressure for the α-, β-, γ-, δ- and κ-phases.

Regarding the evolution of the gap, in Fig. 14 it can be seen that in most phases *E*_g_ decreases under compression, as also happens for ZnV_2_O_6_.^7^ The pressure dependence of *E*_g_ in the different phases can be described as nearly linear function, with the only exception of the triclinic γ-phase which shows a non-linear behavior caused by the previously described band crossing. The pressure coefficients at zero pressure (d*E*_g_/d*P*) are summarized in Table 10. In all the phases, except in the κ-phase the band-gap decreases with pressure. In the α-, β-, and δ-phase the pressure coefficients are similar, but in the γ-phase the pressure coefficient doubles the value of the same parameter in the other three phases. In contrast, in the κ-phase the pressure coefficient has not only the opposite sign (the gap opens under compression), but the absolute value is very small. The closing of the gap with pressure in most of the polymorphs is an indication of the enhancement under compression of the hybridization between V 3d and O 2p orbitals. In the case of the κ-phase the band-gap increases under pressure because of the increase of repulsion between bonding and antibonding states. Such repulsion is what makes this phase to be the one with the largest band gap. In the pressure-range of this study, Zn_2_V_2_O_7_ is a wide band-gap material with an ultraviolet band gap.

Since the different polymorphs of Zn_2_V_2_O_7_ have very different compressibilities (see Table 7), in addition of comparing changes induced by pressure in the band-gap energy, it is interesting to compare changes of the band-gap energy with volume changes. This can be done by introducing a parameter equivalent to the Grüneisen parameter, which we will name as band-gap Grüneisen parameter (*γ*_gap_) and it is defined as 
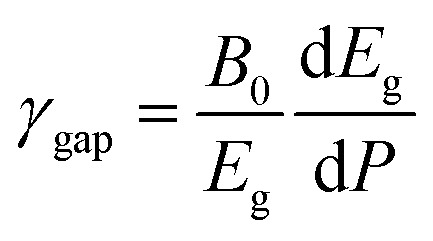
, where *B*_0_ is the bulk modulus. This parameter describes the effect that changing the volume of the crystal lattice has on band-gap energy. The values obtained for *γ*_gap_ are given in Table 10. There is can be seen that influence of volume change in the band-gap energy decrease following the sequence γ → δ → β → α → κ. The *γ*_gap_ value obtained for the κ-phase is comparable with values from orthovanadates (*e.g.* CrVO_4_ and PrVO_4_).^49,50^ The larger sensitivity of the band-gap of other phases of Zn_2_V_2_O_7_ to volume changes in comparison with the κ-phase and other vanadates is an indication that pressure considerably affect orbital hybridization in the α-, β-, γ-, and δ-phase of Zn_2_V_2_O_7_.

## 4. Conclusions

In this work by means of density-functional theory calculations we have studied the high-pressure behavior of zinc pyrovanadate (Zn_2_V_2_O_7_). After considering three different functionals we have found that B3LYP is the one that describe better the crystal structure of Zn_2_V_2_O_7_. Our calculations predict the existence of three phase transitions for pressure smaller than 10 GPa, which contributes to the understanding of previous experimental findings.^7^ The predicted phase transition sequence is α–γ–κ being all the phases in the structural sequence either monoclinic or triclinic. The β-phase found in experiments is very close in enthalpy to the γ-phase. The presence of kinetic barriers has been proposed as the possible reason of the existence of β-Zn_2_V_2_O_7_ as an intermediate phase between the α and γ polymorphs. The compressibility of the different phases has been also studied, being found that Zn_2_V_2_O_7_ is much more compressible than other vanadates. In addition, the response to pressure is found to be non-isotropic. For the different polymorphs, the anisotropy has been discussed in detail, being the principal compression axis determined. Finally, the band-structure and electronic density of states of the different phase have been obtained. All the phases of Zn_2_V_2_O_7_ are found to have a large band gap. Not only the band-gap energy but its pressure dependence is reported. Explanations for the observed phenomena are provided together with a comprehension with previous studies in Zn_2_V_2_O_7_ and related compounds.

## Author contributions

All authors have contributed equally to this work.

## Conflicts of interest

There are no conflicts to declare.

## Supplementary Material

## References

[cit1] Ashrafi S., Mousavi-Kamazani M., Zinatloo-Ajabshir S., Asgharic A. (2020). Novel sonochemical synthesis of Zn_2_V_2_O_7_ nanostructures for electrochemical hydrogen storage. Int. J. Hydrogen Energy.

[cit2] Sameie H., Sabbagh Alvani A. A., Naseri N., Du S., Rosei F. (2018). First-principles study on ZnV_2_O_6_ and Zn_2_V_2_O_7_: Two new photoanode candidates for photoelectrochemical water oxidation. Ceram. Int..

[cit3] Liu H., Cui Y. (2018). Microwave-assisted hydrothermal synthesis of hollow flower-like Zn_2_V_2_O_7_ with enhanced cycling stability as electrode for lithium ion batteries. Mater. Lett..

[cit4] Li Y. D., Teng Y. F., Zhang Z. Q., Feng Y., Xue P., Tong W. M., Liu X. Y. (2017). Microwave-assisted synthesis of novel nanostructured Zn_3_(OH)_2_V_2_O_7_·2H_2_O and Zn_2_V_2_O_7_ as electrode materials for supercapacitors. New J. Chem..

[cit5] Diaz-Anichtchenko D., Turnbull R., Bandiello E., Anzellini S., Errandonea D. (2020). High-Pressure Structural Behavior and Equation of State of Kagome Staircase Compound, Ni_3_V_2_O_8_. Crystals.

[cit6] Chen Z., Huang W., Lu D., Zhao R., Chen H. (2013). Hydrothermal synthesis and electrochemical properties of crystalline Zn_2_V_2_O nanorods. Mater. Lett..

[cit7] Díaz-Anichtchenko D., Santamaria-Perez D., Marqueño T., Pellicer-Porres J., Ruiz-Fuertes J., Ribes R., Ibañez J., Achary S. N., Popescu C., Errandonea D. (2020). Comparative study of the high-pressure behavior of ZnV_2_O_6_, Zn_2_V_2_O_7_ and Zn_3_V_2_O_8_. J. Alloys Compd..

[cit8] Errandonea D. (2020). High pressure crystal structures of orthovanadates and their properties. J. Appl. Phys..

[cit9] Beltrán A., Gracia L., Andrés J. (2019). Polymorphs of ZnV_2_O_6_ under Pressure: A First-Principle Investigation. J. Phys. Chem. C.

[cit10] Lopez-Moreno S., Errandonea D., Rodríguez-Hernández P., Muñoz A. (2015). Polymorphs of CaSeO_4_ under Pressure: A First-Principles Study of Structural, Electronic, and Vibrational Properties. Inorg. Chem..

[cit11] Benmakhlouf A., Errandonea D., Bouchenafa M., Maabed S., Bouhemadou A., Bentabet A. (2017). New pressure-induced polymorphic transitions of anhydrous magnesium sulfate. Dalton Trans..

[cit12] Errandonea D., Gracia L., Lacomba-Perales R., Polian A., Chervin J. C. (2013). Compression of scheelite-type SrMoO_4_ under quasi-hydrostatic conditions: Redefining the high-pressure structural sequence. J. Appl. Phys..

[cit13] Yan Y., Yu Y., Wu D., Yang Y., Cao Y. (2016). TiO_2_/vanadate (Sr_10_V_6_O_25_, Ni_3_V_2_O_8_, Zn_2_V_2_O_7_) heterostructured photocatalysts with enhanced photocatalytic activity for photoreduction of CO_2_ into CH_4_. Nanoscale.

[cit14] González-Rivera Y. A., Meza-Rocha A. N., Aquino-Meneses L., Jiménez-Sandoval S., Rubio-Rosas E., Caldiño U., Álvarez E., Zelaya-Angel O., Toledo-Solano M., Lozada-Morales R. (2016). Photoluminescent and electrical properties of novel Nd^3+^ doped ZnV_2_O_6_ and Zn_2_V_2_O_7_. Ceram. Int..

[cit15] Guan Y., Cheng Y., Huang Y., Tsuboi T., Huang W., Cai P., Seo H. J. (2014). Spectral conversion from ultraviolet to near infrared in Yb^3+^-doped pyrovanadate Zn_2_V_2_O_7_ Particles. J. Am. Ceram. Soc..

[cit16] Kuang S. P., Menga Y., Liua Y., Wua Z. C., Zhao L. S. (2013). A new self-activated yellow emitting phosphor Zn_2_V_2_O_7_ for white LED. Optik.

[cit17] Dovesi R. , Saunders V. R., Roetti C., Orlando R., Zicovich-Wilson C. M., Pascale F., Civalleri B., Doll K., Harrison N. M., Bush I. J., D'Arco P., Llunell M., Causà M. and Noë Y., CRYSTAL14 User's Manual, University of Torino, Torino, 2014

[cit18] Becke A. D. (1993). Density-functional thermochemistry. III. The role of exact exchange. J. Chem. Phys..

[cit19] Lee C., Yang W., Parr R. G. (1988). Development of the Colle-Salvetti correlation-energy formula into a functional of the electron density. Phys. Rev. B.

[cit20] Heyd J., Scuseria G. E., Erratum Ernzerhof M. (2003). Hybrid functionals based on a screened Coulomb potential. J. Chem. Phys..

[cit21] Perdew J. P., Wang Y. (1992). Accurate and simple analytic representation of the electron-gas correlation energy. Phys. Rev. B.

[cit22] http://www.crystal.unito.it/basis-sets.php/

[cit23] Errandonea D., Manjón F. J. (2008). Pressure effects on the structural and electronic properties of ABX_4_ scintillating crystals. Prog. Mater. Sci..

[cit24] Gopal R., Calvo C. (1973). Crystal structure of α – Zn_2_V_2_O_7_. Can. J. Chem..

[cit25] Au P. K. L., Calvo C. (1967). Crystal structure of Cd_2_V_2_O_7_. Can. J. Chem..

[cit26] Gopal R., Calvo C. (1974). Crystal structure of magnesium divanadete Mg_2_V_2_O_7_. Acta Crystallogr. B.

[cit27] Quarton M., Angenault J., Rimsky A. (1973). Structure cristalline de alpha-Hg_2_V_2_O_7_. Acta Crystallogr. B.

[cit28] Shannon R. D., Calvo C. (1973). Refinement of the Crystal Structure of Synthetic Chervetite, Pb_2_V_2_O_7_. Can. J. Chem..

[cit29] Vedernikov A. A., Velikodnyi Y. A., Lliyukhin V. V., Trunov V. K. (1982). Crystal structure of strontium diorthovanadate Hg_2_V_2_O_7_. Sov. Phys. Dokl..

[cit30] Nielsen U. G., Jakobsen H. J., Skibsted J., Norby P. (2001). Crystal structure of α-Mg_2_V_2_O_7_ from synchrotron X-ray powder diffraction and characterization by 51V MAS NMR spectroscopy. Dalton Trans..

[cit31] Freysoldt C., Grabowski B., Hickel T., Neugebauer J., Kresse G., Janotti A., van de Walle C. G. (2014). First-principles calculations for point defects in solids. Rev. Mod. Phys..

[cit32] Grimme S. (2006). Semiempirical GGA-type density functional constructed with a long-range dispersion correction. Journal Computing Chemistry.

[cit33] Birch F. (1952). Elasticity and constitution of the Earth's interior. J. Geophys. Res..

[cit34] Bandiello E., Errandonea D., Pellicer-Porres J., Garg A. B., Rodriguez-Hernandez P., Muñoz A., Martinez-Garcia D., Rao R., Popescu C. (2018). Effect of High pressure on the crystal structure and vibrational properties of olivine-type LiNiPO_4_. Inorg. Chem..

[cit35] Errandonea D., Gomis O., Santamaría-Perez D., García-Domene B., Muñoz A., Rodríguez-Hernández P., Achary S. N., Tyagi A. K., Popescu C. (2015). Exploring the high-pressure behaviour of the three known polymorphs of BiPO_4_: Discovery of a new polymorph. J. Appl. Phys..

[cit36] Krasnenko T. I., Zubkov V. G., Tyutyunnik A. P., Zolotukhina L. V., Vasyutinskaya E. F. (2003). Crystal structure of β-Zn_2_V_2_O_7_. Crystallogr. Rep..

[cit37] Gonzalez-Platas J., Lopez-Moreno S., Bandiello E., Bettinelli M., Errandonea D. (2020). Precise Characterization of the Rich Structural Landscape Induced by Pressure in Multifunctional FeVO_4_. Inorg. Chem..

[cit38] Ouahrani T., Medjdoub F. Z., Gueddida S., Lobato Fernandez A., Franco R., Benkhettou N. E., Badawi M., Liang A., Gonzalez J., Errandonea D. (2021). Understanding the pressure effect on the elastic, electronic, vibrational, and bonding properties of the CeScO_3_ perovskite. J. Phys. Chem. C.

[cit39] Knight K. S. (2010). Analytical expressions to determine the isothermal compressibility tensor and the isobaric thermal expansion tensor for monoclinic crystals: application to determine the direction of maximum compressibility in jadeite. Physical Chemical Minerals.

[cit40] Turnbull R., Errandonea D., Cuenca-Gotor V. P., Sans J. A., Gomis O., Gonzalez A., Rodríguez-Hernandez P., Popescu C., Bettinelli M., Mishra K. K., Manjon F. J. (2021). Experimental and theoretical study of dense YBO_3_ and the influence of non-hydrostaticity. J. Alloys Compd..

[cit41] Cliffe M. J., Goodwin A. L. (2012). PASCAL: a principal axis strain calculator for thermal expansion and compressibility determination. J. Appl. Crystallogr..

[cit42] Kimizuka H., Ogata S., Shibutani Y. (2005). High-pressure elasticity and auxetic property of α-cristobalite. Mater. Trans..

[cit43] Adachi K., Ogi H., Takeuchi N., Nakamura N., Watanabe H., Ito T., Ozaki Y. (2018). Unusual elasticity of monoclinic β-Ga_2_O_3_. J. Appl. Phys..

[cit44] Singh J., Sharma V. K., Kanchana V., Vaitheeswaran G., Errandonea D. (2020). High-pressure structural, lattice dynamics, and electronic properties of beryllium aluminate studied from first-principles theory. Mater. Today Commun..

[cit45] Hoppe R., Voigt S., Glaum H., Kissel J., Müller H. P., Bernet K. (1989). A new route to charge distributions in ionic solids. J. Less Common. Met..

[cit46] Botella P., Errandonea D., Garg A. B., Rodriguez-Hernandez P., Muñoz A., Achary S. N., Vomiero A. (2019). High-pressure characterization of the optical and electronic properties of InVO_4_, InNbO_4_, and InTaO_4_. SN Applied Sciences.

[cit47] Schira R., Latouche C. (2020). DFT an hybrid-DFT calculations on the electronic properties of vanadate materials: theory meets experiments. New J. Chem..

[cit48] Errandonea D., Garg A. B. (2018). Recent progress on the characterization of the high-pressure behaviour of AVO_4_ orthovanadates. Prog. Mater. Sci..

[cit49] Botella P., López-Moreno S., Errandonea D., Manjón F. J., Sans J. A., Vie D., Vomiero A. (2020). High-pressure characterization of multifunctional CrVO_4_. J. Phys. Condens. Matter.

[cit50] Bandiello E., Popescu C., Lora da Silva E., Sans J. A., Errandonea D., Bettinelli M. (2020). PrVO_4_ under high pressure: Effects on structural, optical, and electrical properties. Inorg. Chem..

